# Mix-and-Match System for the Enzymatic Synthesis of Enantiopure Glycerol-3-Phosphate-Containing Capsule Polymer Backbones from *Actinobacillus pleuropneumoniae*, *Neisseria meningitidis*, and *Bibersteinia trehalosi*

**DOI:** 10.1128/mBio.00897-21

**Published:** 2021-05-26

**Authors:** Christa Litschko, Insa Budde, Monika Berger, Andrea Bethe, Julia Schulze, E. Alberto Alcala Orozco, Reza Mahour, Peter Goettig, Jana Indra Führing, Thomas Rexer, Rita Gerardy-Schahn, Mario Schubert, Timm Fiebig

**Affiliations:** a Institute of Clinical Biochemistry, Hannover Medical School, Hannover, Germany; b Max Planck Institute for Dynamics of Complex Technical Systems, Bioprocess Engineering, Magdeburg, Germany; c Department of Biosciences, University of Salzburg, Salzburg, Austria; d Fraunhofer International Consortium for Anti-Infective Research (iCAIR), Hannover, Germany; GSK Vaccines

**Keywords:** enzymatic synthesis, capsule, glycoconjugate vaccine, antibiotic resistance, glycerol, polysaccharides, teichoic acid, animal health, TagF-like polymerases, veterinary vaccine development, glycosyltransferase, recombinant protein expression, NMR

## Abstract

Capsule polymers are crucial virulence factors of pathogenic bacteria and are used as antigens in glycoconjugate vaccine formulations. Some Gram-negative pathogens express poly(glycosylglycerol phosphate) capsule polymers that resemble Gram-positive wall teichoic acids and are synthesized by TagF-like capsule polymerases. So far, the biotechnological use of these enzymes for vaccine developmental studies was restricted by the unavailability of enantiopure CDP-glycerol, one of the donor substrates required for polymer assembly. Here, we use CTP:glycerol-phosphate cytidylyltransferases (GCTs) and TagF-like polymerases to synthesize the poly(glycosylglycerol phosphate) capsule polymer backbones of the porcine pathogen Actinobacillus pleuropneumoniae, serotypes 3 and 7 (*App*3 and *App*7). GCT activity was confirmed by high-performance liquid chromatography, and polymers were analyzed using comprehensive nuclear magnetic resonance studies. Solid-phase synthesis protocols were established to allow potential scale-up of polymer production. In addition, one-pot reactions exploiting glycerol-kinase allowed us to start the reaction from inexpensive, widely available substrates. Finally, this study highlights that multidomain TagF-like polymerases can be transformed by mutagenesis of active site residues into single-action transferases, which in turn can act in *trans* to build-up structurally new polymers. Overall, our protocols provide enantiopure, nature-identical capsule polymer backbones from *App*2, *App*3, *App*7, *App*9, and *App*11, Neisseria meningitidis serogroup H, and Bibersteinia trehalosi serotypes T3 and T15.

## INTRODUCTION

Multidrug-resistant bacteria are a threat to public health and the implementation of novel therapeutic or prophylactic approaches is challenging (1). One reason for increasing resistance is the use of broad-band antibiotics in animal husbandry (2). Actinobacillus pleuropneumoniae, a pig-specific, Gram-negative, encapsulated pathogen, and causative agent of highly contagious porcine pleuropneumonia, presents an economic burden for the swine industry worldwide and is typically treated with antibiotics (3). Although vaccines based on A. pleuropneumoniae toxins, attenuated strains, and subunit vaccines exist, they fail to protect against transmission (3, 4). According to a recent study (5), in herds with a high prevalence of clinical disease, the administration of a high-efficacy vaccine would be economically superior to the application of medicine. Thus, the implementation of new vaccination strategies against A. pleuropneumoniae could prevent the use of antibiotics and be of major advantage for global health.

**FIG 1:**
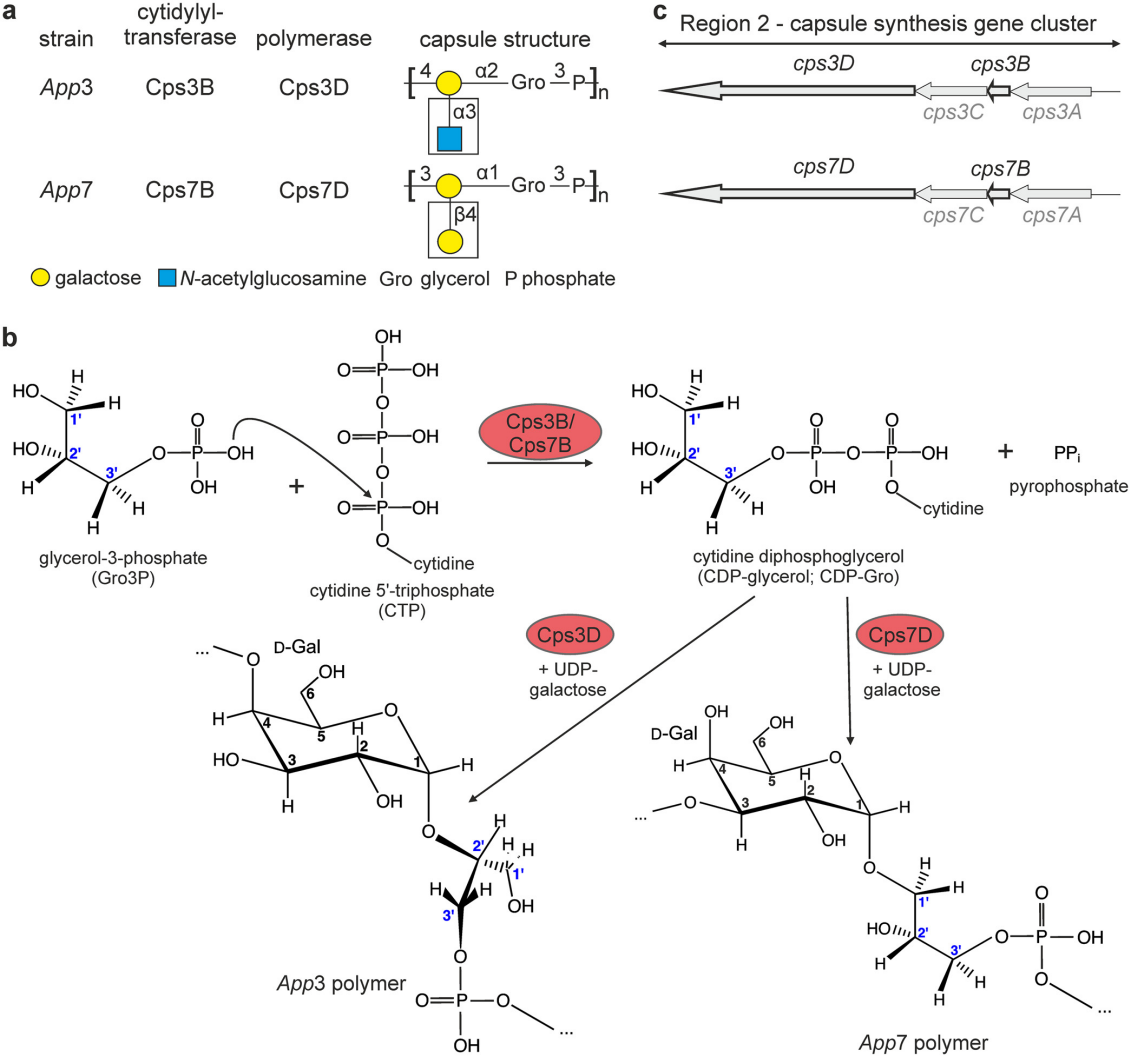
Capsule synthesis in *App*3 and *App*7. (a) Cytidylyltransferases (GCTs), polymerases, and capsule polymer structures of *App*3 and *App*7. Modifications (white boxes) of the linear capsule polymer backbones are introduced by separate and as-yet-unidentified enzymes. (b) Reactions catalyzed by the gene products Cps3B/Cps7B and Cps3D/Cps7D. (c) Schematic representation of region 2 of the capsule gene clusters of *App*3 and *App*7.

Most effective antibacterial vaccine formulations contain glycoconjugates made of capsule polymers coupled to carrier proteins (6). For the conventional production of glycoconjugate vaccines, these polymers are obtained through mass cultivation of the pathogen, which requires highest biosafety standards (7), causes considerable costs, and thus prevents broad application in animal husbandry. *In vivo*, capsule polymers are synthesized by capsule polymerases before they are exported outside the cell, where they form a thick layer that protects the pathogen from the immune system of its host (8). In recent years, capsule polymerases have become attractive synthesis tools for both *in vivo* and *in vitro* synthesis systems, because their use is pathogen-free, stereoselective and regioselective, highly efficient, and even allows the buildup of large and complex structures unobtainable through chemical synthesis (9, 10). Recently, we identified a new family of capsule polymerases, the TagF-like capsule polymerase family, which is abundant in Gram-negative human and animal pathogens expressing a group 2 capsule such as Neisseria meningitidis, A. pleuropneumoniae, Haemophilus influenzae, Bibersteinia trehalosi, and Escherichia coli (11). Members of this family represent multidomain enzymes that generate complex phosphate-containing capsules. One subset synthesizes poly(oligosaccharide phosphate), a second subset assembles poly(glycosylpolyol phosphate), which is similar to wall teichoic acid type II (11). TagF-like capsule polymerases have a modular architecture, whose common denominator is the TagF-like fold. It transfers either glycerol- or hexose/N-acetylhexosamine-phosphate and can be paired with an N-terminal GT-A folded domain adding a hexose/N-acetylhexosamine in beta-linkage or with a C-terminal GT-B folded domain adding a hexose/N-acetylhexosamine in alpha-linkage. The latter subset includes the enzymes Cps3D and Cps7D from A. pleuropneumoniae serotypes 3 and 7 (*App*3 and *App*7), respectively, which generate the poly(glycosylglycerol phosphate) structures [→4)-α-Gal-(1→2)-Gro-(3-PO_4_^–^] and [→3)-α-Gal-(1→1)-Gro-(3-PO_4_^–^] starting from UDP-galactose (UDP-Gal) and CDP-glycerol (CDP-Gro) (Fig. 1). Both enzymes were shown to incorporate *sn*-glycerol-1-phosphate (Gro1P) and *sn*-glycerol-3-phosphate (Gro3P) (enantiomers due to chirality at C2) from a racemic mixture of CDP-Gro into their polymer product (11), even though *in vivo*, the *App*7 polymer backbone was found to exclusively contain Gro3P (12). The stereochemistry of glycerol in the *App*3 polymer has not yet been analyzed (13).

In bacteria, CDP-Gro is usually synthesized by CTP:*sn*-glycerol-3-phosphate cytidylyltransferases (GCTs) from the substrates CTP and Gro3P (Fig. 1b) (14). The genes *cps3B* and *cps7B*, localized in the capsule gene cluster of A. pleuropneumoniae serotypes 3 and 7 (Fig. 1c), respectively, were predicted to encode these enzymes (15). Enantiopure CDP-Gro is commercially not available and to the best of our knowledge, GCTs have never been used to supply CDP-Gro for preparative-scale syntheses of polymers. For this study, we designed a novel enzymatic cascade using GCTs and TagF-like polymerases and hypothesize that it can provide enantiopure poly(glycosylglycerol-phosphate) at a preparative-scale for vaccine development.

## RESULTS

### Identification of GCTs in *App*3 and *App*7.

**FIG 2:**
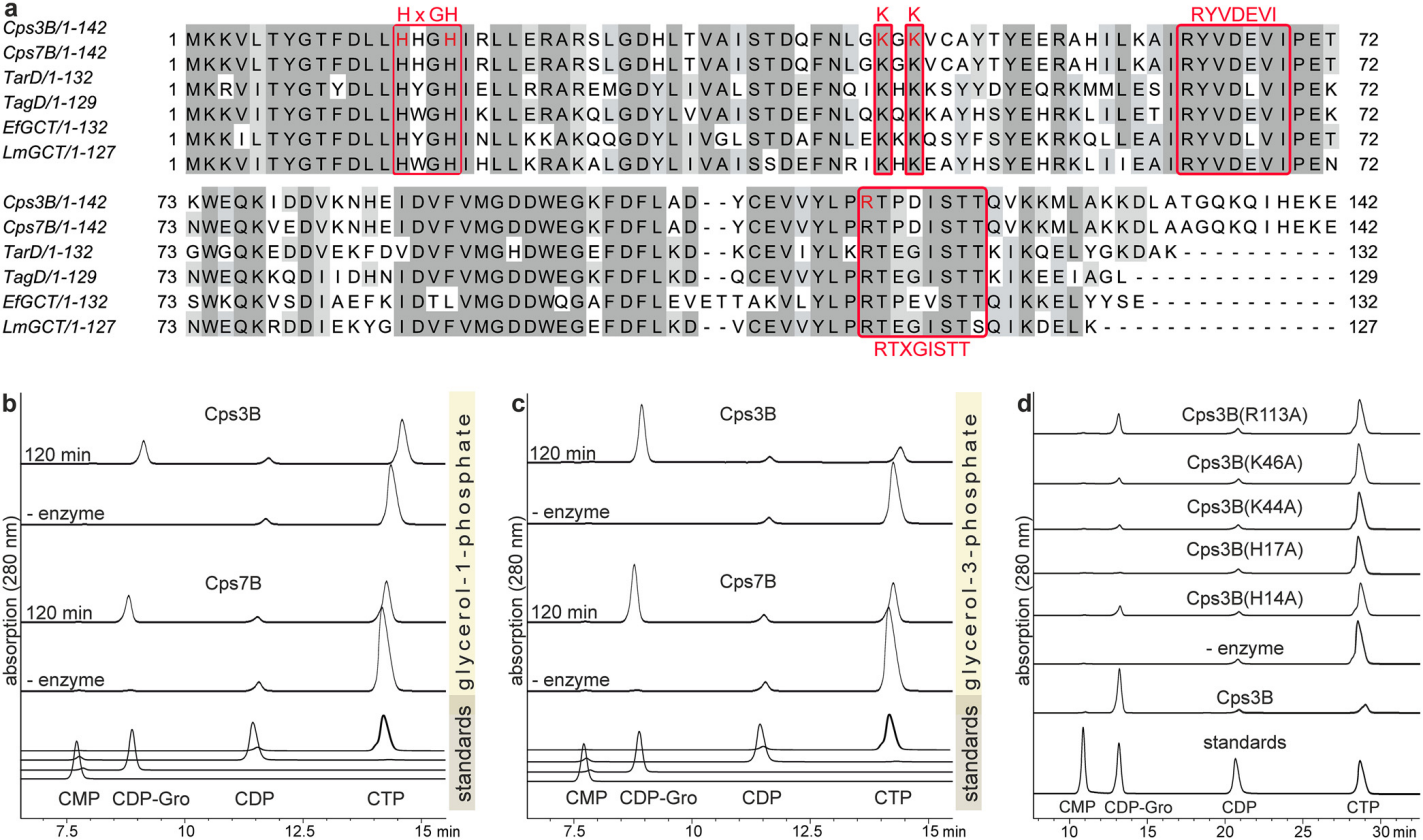
Alignment of different GCT amino acid sequences and activity of Cps3B and Cps7B. (a) Amino acid sequence alignment of Cps3B (*App*3, GenBank ABY70165.1), Cps7B (*App*7, GenBank ACE62293.1), TarD (S. aureus, GenBank CAA60586.1), TagD (B. subtilis, GenBank AAA22843.1), GCT (E. faecalis, GenBank AAO80975.1), and GCT (L. monocytogenes, GenBank AE016830). Identical amino acids are highlighted in gray, and conserved motifs and residues are boxed (in red). Residues mutated in Cps3B in this study are indicated by red text. (b and c) Substrate consumption in Cps3B and Cps7B reactions in the presence of Gro1P (b) or Gro3P (c) analyzed via HPLC-AEC coupled to UV detection. (d) Activity of Cps3B mutants analyzed by HPLC-AEC.

Centerpiece of the envisioned synthesis cascade is a suitable GCT for the supply of enantiopure CDP-Gro. A number of bacterial GCTs were previously characterized and showed activity *in vitro*, e.g., the GCTs from Bacillus subtilis (16), Staphylococcus aureus (17, 18), Enterococcus faecalis, and Listeria monocytogenes (19). Apart from two exceptions (20), all GCTs belong to a larger family of cytidylyltransferases comprising GCT, CTP:phosphoethanolamine cytidylyltransferase (ECT), CTP:2-C-methyl-d-erythritol-4-phosphate cytidylyltransferase (CMS), and CTP:phosphocholine cytidylyltransferase (CCT) (21). Members of this family contain two highly conserved sequences (Fig. 2a) involved in the binding of CTP: the HxGH motif, also identified in class I aminoacyl-tRNA synthetases, and the RTXGISTT motif, which is unique to the cytidylyltransferase family (22). A third conserved motif, RYVDEVI, is part of the dimer interface (14). In TagD from Bacillus subtilis, several residues, such as His14 and His17 of the HxGH motif and Arg113 of the RTEGISTT motif, were shown to be essential for catalytic function (22). Two lysine residues, Lys44 and Lys46, were shown to interact with the negatively charged phosphates of CDP-Gro (23). The genes *cps3B* and *cps7B* from *App*3 and *App*7, respectively, were predicted to encode glycerol-3-phosphate cytidylyltransferases (15). They share 97% amino acid sequence identity with each other, ∼70% sequence identity with TagD from Bacillus subtilis, ∼61% identity with TarD from Staphylococcus aureus, ∼60% identity with GCT from Enterococcus faecalis, and ∼67% identity with GCT from Listeria monocytogenes. An alignment demonstrates that Cps3B and Cps7B indeed harbor all characteristic motives of the cytidylyltransferase family (Fig. 2a).

To corroborate the putative GCT activity of Cps3B and Cps7B, we cloned, expressed, and purified both proteins. Activity was assessed by incubating recombinant Cps3B and Cps7B with their putative donor substrates CTP and GroP (both Gro1P and Gro3P were tested as potential substrates). We used high-performance liquid chromatography–anion-exchange chromatography (HPLC-AEC) coupled to UV detection (280 nm) to monitor the consumption and production of nucleotide substrates and products, respectively. Interestingly, CTP was consumed and CDP-Gro was produced in the presence of either GroP enantiomer (Fig. 2b and c). A precise quantitative assessment of GCT activity was beyond the scope of this study. Nevertheless, HPLC-AEC data indicate (Fig. 2b and c) that CTP consumption and CDP-Gro production were higher in reaction mixtures containing Gro3P, suggesting a preference for this substrate. To corroborate that the enzymes belong to the cytidylyltransferase family, residues presumably critical for activity of Cps3B were mutated to alanine. All mutants displayed reduced activity, indicated by low or absent CDP-Gro production and low CTP consumption (Fig. 2d).

### Production of enantiopure *App*3 and *App*7 capsule backbones in one-pot reactions.

**FIG 3:**
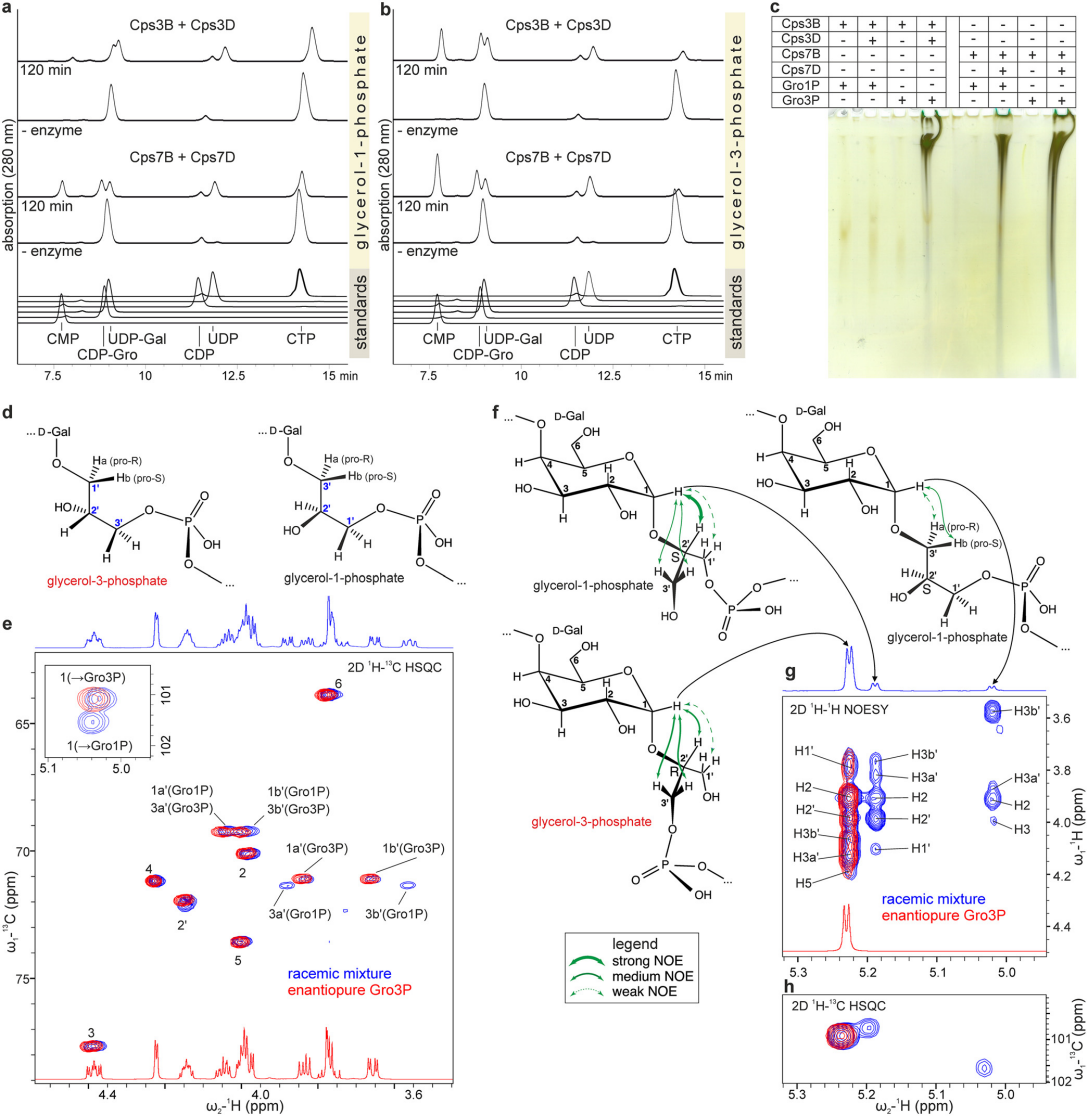
Synthesis of enantiopure *App*3 and *App*7 polymers. (a and b) HPLC-AEC analysis of one-pot reaction mixtures containing GCTs, polymerases, and Gro1P or Gro3P, as indicated. Slight differences in retention time can result from different sample compositions (e.g., with or without enzyme). (c) Corresponding Alcian blue/silver-stained PAGE samples for visualization of synthesized polymer. (d) Chemical structures of Gro1P- and Gro3P-containing repeating units synthesized by Cps7D. (e) 2D ^1^H-^13^C HSQC spectra of polymer synthesized by Cps7D from racemic CDP-Gro (blue correlations) and enantiopure CDP-3-Gro (red correlations). (f) Chemical structures of Gro1P- and Gro3P-containing repeating units synthesized by Cps3D. Green arrows indicate NOEs (nuclear Overhauser effects, see legend). (g) 2D ^1^H-^1^H NOESY spectra of polymer synthesized by Cps3D from racemic CDP-Gro (blue correlations) and enantiopure (red correlations) CDP-3-Gro. (h) Corresponding 2D ^1^H-^13^C HSQC showing anomeric correlations. Reference spectra of racemic polymers were obtained from an earlier study (11).

Enzymatic one-pot reactions are powerful tools for the generation of complex biomolecules (24). Here, we investigated one-pot reactions of Cps3B+Cps3D and Cps7B+Cps7D. Reactions were performed in the presence of either GroP enantiomer, and UDP-Gal was added as second donor substrate for the polymerases (see reaction in Fig. 1b). Nucleotide substrate and product composition was analyzed prior to the addition of enzymes and after 120 min of incubation. The chromatograms demonstrate the consumption of CTP and the production of CDP-Gro (Fig. 3a and b). As expected, the nucleotide products CMP and UDP were detected, resulting from CDP-Gro and UDP-Gal consumption during polymer assembly. Again, the enzymes appear to prefer Gro3P over Gro1P, as indicated by higher levels of CMP and higher consumption of CTP. In agreement with this, Alcian blue/silver-stained PAGE gels corroborated that both polymerases were able to utilize Gro3P, while only Cps7D was able to assemble polymer from Gro1P (Fig. 3c). UDP, present in reaction mixtures containing Cps3D and Gro1P, results from enzyme-facilitated hydrolysis of UDP-Gal, which has been previously reported for TagF-like polymerases lacking appropriate donor and acceptor substrates (11, 25).

Despite the above demonstrated inability of Cps3D to utilize CDP-1-Gro, we previously showed that both Cps3D and Cps7D incorporated both enantiomers from a racemic mixture of CDP-Gro (11), creating an artificial polymer containing both GroP enantiomers. The nuclear magnetic resonance (NMR) chemical shifts of the resulting polymers (Fig. 3e, g, and h, blue correlations) diverge from the chemical shifts reported for the native polymers, especially for the atoms contributing to the glycosidic linkages (11). In the native *App*7 polymer backbone, the glycosidic linkage is established between C1 of Gal and C1 of Gro3P (Fig. 3d, left) (12). In contrast, in the artificial repeating unit containing Gro1P, the glycosidic linkage connects C1 of Gal to C3 of Gro1P (Fig. 3d, right). As a result, the C1/H1 cross-peak observed for Gal in the artificial repeating unit is shifted downfield in the two-dimensional (2D) ^1^H-^13^C heteronuclear single quantum coherence (HSQC) spectrum (Fig. 3e, zoomed-in section) and cross-peaks for C3′/H3a′ and C3′/H3b′ of Gro appear in addition to the cross-peaks observed for C1′/H1a′ and C1′/H1b′ (Fig. 3e, blue spectrum). To investigate whether this heterogeneity disappears in polymers assembled from CDP-3-Gro using Cps7B and Cps7D, the one-pot reaction was upscaled to yield 5 mg of polymer, and 2D NMR analysis of the product was performed after purification via preparative AEC. Importantly, the ^1^H and ^13^C chemical shifts deduced from the 2D ^1^H-^13^C HSQC spectrum (Fig. 3e, red spectrum, and Table 1) and confirmed by 2D ^1^H-^1^H COSY (correlation spectroscopy), ^1^H-^1^H TOCSY (total correlation spectroscopy), and ^1^H-^31^P HMBC (heteronuclear multiple bond correlation) spectra (data not shown) are in perfect agreement with the chemical shifts reported for the natural polymer (12) and lack any heterogeneity.

**TABLE 1:** ^1^H and ^13^C chemical shifts of the Gro3P-containing polymers referenced to DSS (2,2-dimethyl-2-silapentanesulfonic acid)

Type	Chemical shifts (ppm)								
	Gal						Gro		
	C1/H1	C2/H2	C3/H3	C4/H4	C5/H5	C6/H6(+H6′)	C1/H1(+H1′)	C2/H2	C3/H3(+H3′)
Cps3D	100.9/5.24	71.2/3.92	71.6/4.01	77.4/4.56	73.5/4.20	63.6/3.77	64.0/3.81	79.9/3.99	67.2/4.14+4.08
Cps7D	101.1/5.04	70.1/3.99	77.7/4.39	71.2/4.23	73.6/4.01	63.9/3.78	71.1/3.84+3.67	71.9/4.15	69.2/4.05+4.01
Cps11D	100.9/5.23	71.1/3.91	71.5/4.01	77.4/4.56	73.5/4.20	63.6/3.78	64.0/3.81	79.9/3.99	67.2/4.14+4.08
Bt188	101.2/5.00	71.2/3.91	71.7/3.99	77.5/4.55	73.5/4.04	63.7/3.78+3.75	71.1/3.82+3.64	72.0/4.12	69.4/4.06+3.98

The incorporation of both GroP enantiomers by Cps3D leads to the formation of three different repeating units appearing as distinct spin systems with well-isolated anomeric signals best visualized by ^1^H-^1^H NOESY (nuclear Overhauser enhancement spectroscopy) and ^1^H-^13^C HSQC (Fig. 3g and h) (11). The most intense and most downfield signal (5.23 ppm) results from the repeating unit in which the glycosidic linkage is formed with O2′ of Gro3P (Fig. 3f, bottom, g, and h, and see Fig. S1 in the supplemental material). When Gro1P is incorporated, the glycosidic linkage is either correctly formed at O2′ (Fig. 3f, top left) or misplaced at O3′ (Fig. 3f, top right). The spin systems resulting from these repeating units are less abundant with well-isolated anomeric signals at 5.19 and 5.02 ppm, respectively. In contrast to the artificial polymer produced from racemic CDP-Gro, the polymer isolated from an upscaled one-pot reaction mixture containing Cps3B and Cps3D, together with enantiopure Gro3P, lacked any heterogeneity and was in perfect agreement with the native *App*3 polymer (13) (Fig. 3g and h, red spectrum, and Table 1). This finding suggests that also the native *App*3 polymer is generated from CDP-3-Gro.

10.1128/mBio.00897-21.2 FIG S1 Stereoview of a repeating unit of a [→4)-α-Gal-(1→2)-Gro-(3-PO_4_^–^] (*App*3) hexamer. The distances between Gal H1 and the surrounding protons in the model are in good agreement to the distances derived from NOE cross-peak intensities from 2D NOESY spectra shown in Fig. 3g (H1-H2′, H1-H3a′, H1-H3b′, all < 3.0 Å) as previously described (Aeschbacher T, Zierke M, Smieško M, Collot M, Mallet J-M, Ernst B, Allain FHT, Schubert M, 2017, Chemistry 23:11598–11610, doi:10.1002/chem.201701866). The model was generated using CarbBuilder (Kuttel MM, Ståhle J, Widmalm G, 2016, J Comput Chem 37:2098–3105, doi:10.1002/jcc.24428) was further refined by a molecular dynamics calculation using Yasara (Krieger E, Vriend G, 2015, J Comput Chem 36:996–1007, doi:10.1002/jcc.23899), and the image was generated using Molmol (Koradi R, Billeter M, Wüthrich K, 1996, J Mol Graph 14:51–5, 29–32, doi:10.1016/0263-7855(96)00009-4). Download FIG S1, PDF file, 0.3 MB. Copyright © 2021 Litschko et al. 2021 Litschko et al. https://creativecommons.org/licenses/by/4.0/ This content is distributed under the terms of the Creative Commons Attribution 4.0 International license.

### GCTs can be combined with polymerases of other serotypes and genera.

TagF-like capsule polymerases can generate a large variety of structures, which are the basis for the serotyping of encapsulated pathogens (11). To investigate whether GCTs from one serotype can be combined with polymerases from other serotypes or other genera, a one-pot reaction mixture containing Cps7B and the TagF-like capsule polymerase Cps11D (*App*11) was set up. In addition, the TagF-like polymerase Bt188 (11), encoded by the as-yet-nonserotyped Bibersteinia trehalosi isolate USDA-ARS-USMARC-188 (26) was investigated. Being a major agent of pneumonia and septicemia in big horn and commercial sheep, B. trehalosi is becoming increasingly important for cattle farming as well (27, 28). Three of the four B. trehalosi serotypes described so far express glycosylgylcerol-phosphate polymers assembled by TagF-like polymerases, making B. trehalosi an ideal target for extending the technology described here.

**FIG 4:**
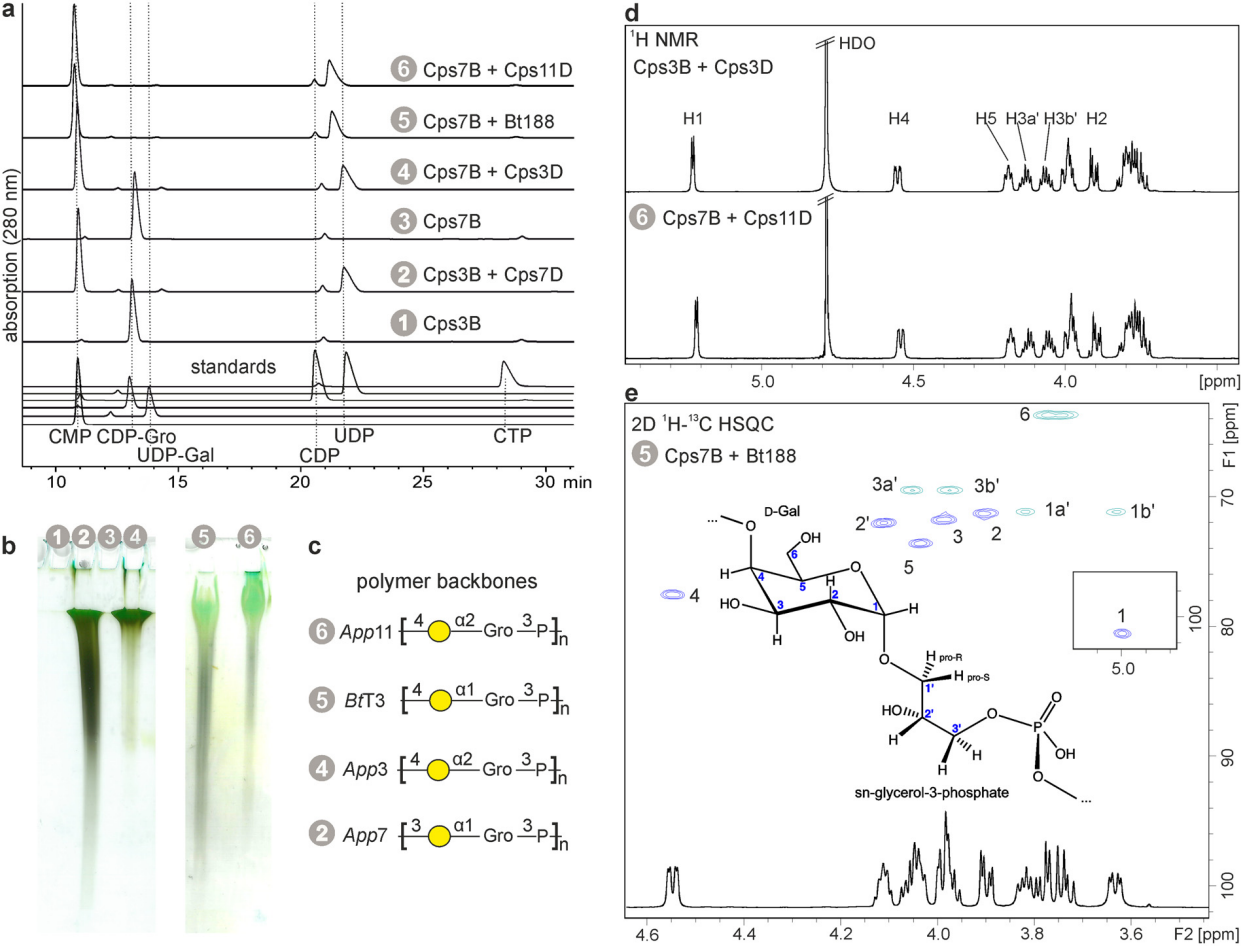
Combining the GCTs Cps3B and Cps7B with polymerases of different serotypes and genera in one-pot reactions. (a) HPLC-AEC of one-pot reaction mixtures containing GCTs and polymerases, as indicated. Reactions 1 and 3 were used as positive controls for CDP-Gro production. (b and c) Corresponding polyacrylamide (PA) gel stained with Alcian blue/silver (b) and polymer structures (c). (d) ^1^H NMR spectra of polymers synthesized in one-pot reaction mixtures containing GCTs and polymerases, as indicated. (e) 2D ^1^H-^13^C HSQC spectrum of the polymer synthesized in a one-pot reaction mixture containing Cps7B and Bt188.

Both polymerases were analyzed by homology modeling in previous studies (11), but enzymatic activity had so far only been confirmed for Cps11D (29). Cps11D was cloned, expressed, and purified as previously described for Cps3D, which generates the same capsule polymer backbone (11). A coding sequence for Bt188 was reverse translated, synthesized, and purified from E. coli expression cultures via its C-terminal His_6_ tag. One-pot reactions combining Cps7B with either Cps11D or Bt188 were incubated overnight, and substrate turnover was analyzed by HPLC-based AEC and via high-percentage PAGE. The combinations Cps3B/Cps7D and Cps7B/Cps3D were tested as well. In all reactions, donor substrates (CTP, CDP-Gro, and UDP-Gal) were completely converted to nucleotide products (CMP and UDP) (Fig. 4a), and polymer could be detected (Fig. 4b). To confirm the identity of the polymers, reactions were scaled up and analyzed by NMR. A ^1^H NMR spectrum was sufficient to demonstrate the identity between the polymers produced by Cps3D and Cps11D (Fig. 4d), corroborating the predicted activity of Cps11D. Importantly, the ^1^H and ^13^C chemical shifts deduced from a ^1^H-^13^C HSQC experiment of the polymer produced by Bt188 were in perfect agreement with published data of the native, de-*O*-acetylated polymer of B. trehalosi serotype T3 (30), indicating that this as-yet-nonserotyped isolate expresses a T3 polymer backbone (Fig. 4e and Table 1). In summary, the above presented experiments demonstrate that Cps3B and Cps7B can be successfully combined with various capsule polymerases in one-pot reactions to yield the desired polymer products.

### Exploiting the modular architecture of TagF-like polymerases.

**FIG 5:**
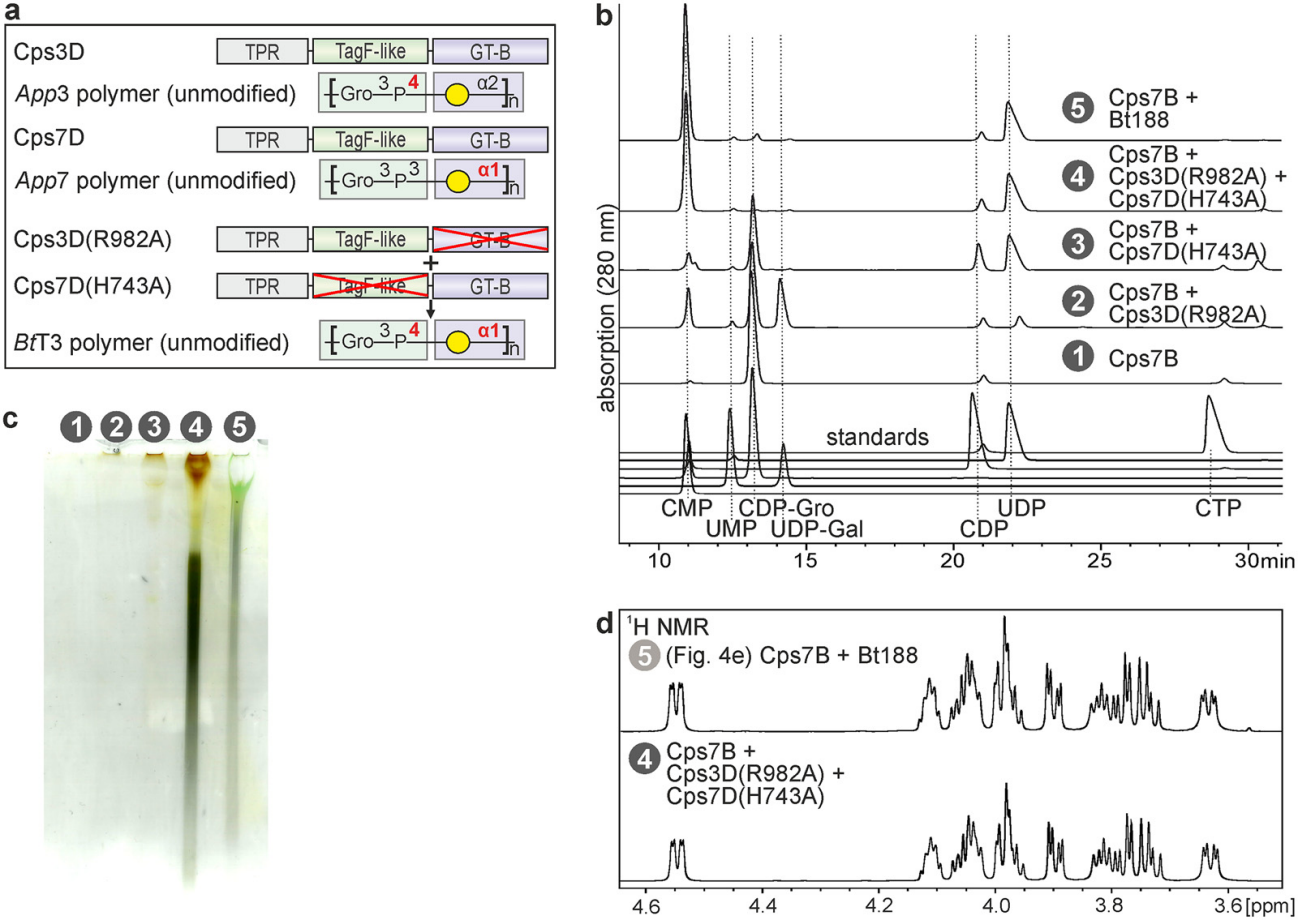
Exploiting the modular architecture of TagF-like polymerases. (a) Domain organization of Cps3D and Cps7D, and the structural composition of their polymer products. Green and violet background colors indicate the domains that transfer the respective moiety. Combining the single-action Cps3D mutant Cps3D(R982A) that transfers Gro3P onto O4 of Gal and the single-action Cps7D mutant Cps7D(H743A) that generates a glycosidic linkage between Gal and O1 of Gro3P yields the polymer backbone of *Bt*T3. (b) HPLC-AEC analysis of one-pot reaction mixtures containing Cps7B and single-action transferases, as indicated. Bt188 was used as a positive control. Single-action transferases alone were used as negative control. Elevated levels of CMP and UDP in samples 2 and 3, respectively, are due to enzyme facilitated hydrolysis catalyzed by the remaining active domain. (c) Corresponding PA gel stained with Alcian blue/silver. (d) ^1^H NMR spectra of the polymer synthesized by Bt188 and the combination of Cps3D(R982A) and Cps7D(H743A).

The modular architecture of TagF-like capsule polymerases is one of the major advantages for exploiting these enzymes as biosynthesis tools. We previously showed that inactivation of one of the two catalytic domains of Cps7D leads to single-action transferases having either Gal-transferase or GroP-transferase activity (11). Combining the respective mutants restores polymerase activity to wild-type levels, corroborating that each domain is independent from the other with regard to activity and folding. To broaden the structural variety of polymers producible by TagF-like capsule polymerases, we investigated whether single-action transferases from different serotypes, e.g., the GroP-transferase activity of Cps3D and the Gal-transferase activity of Cps7D, could be combined to generate new, non-*App*3/7 structures like the polymer backbone of *B. trehalosi* serotype T3 (*Bt*T3) (Fig. 5a). A one-pot reaction mixture containing both single-action transferases was set up and analyzed by HPLC-AEC and PAGE. Indeed, substrate consumption was complete and polymer identical to that synthesized by Bt188 was produced (Fig. 5b and c) as shown by ^1^H NMR (Fig. 5d).

### Solid-phase synthesis.

**FIG 6:**
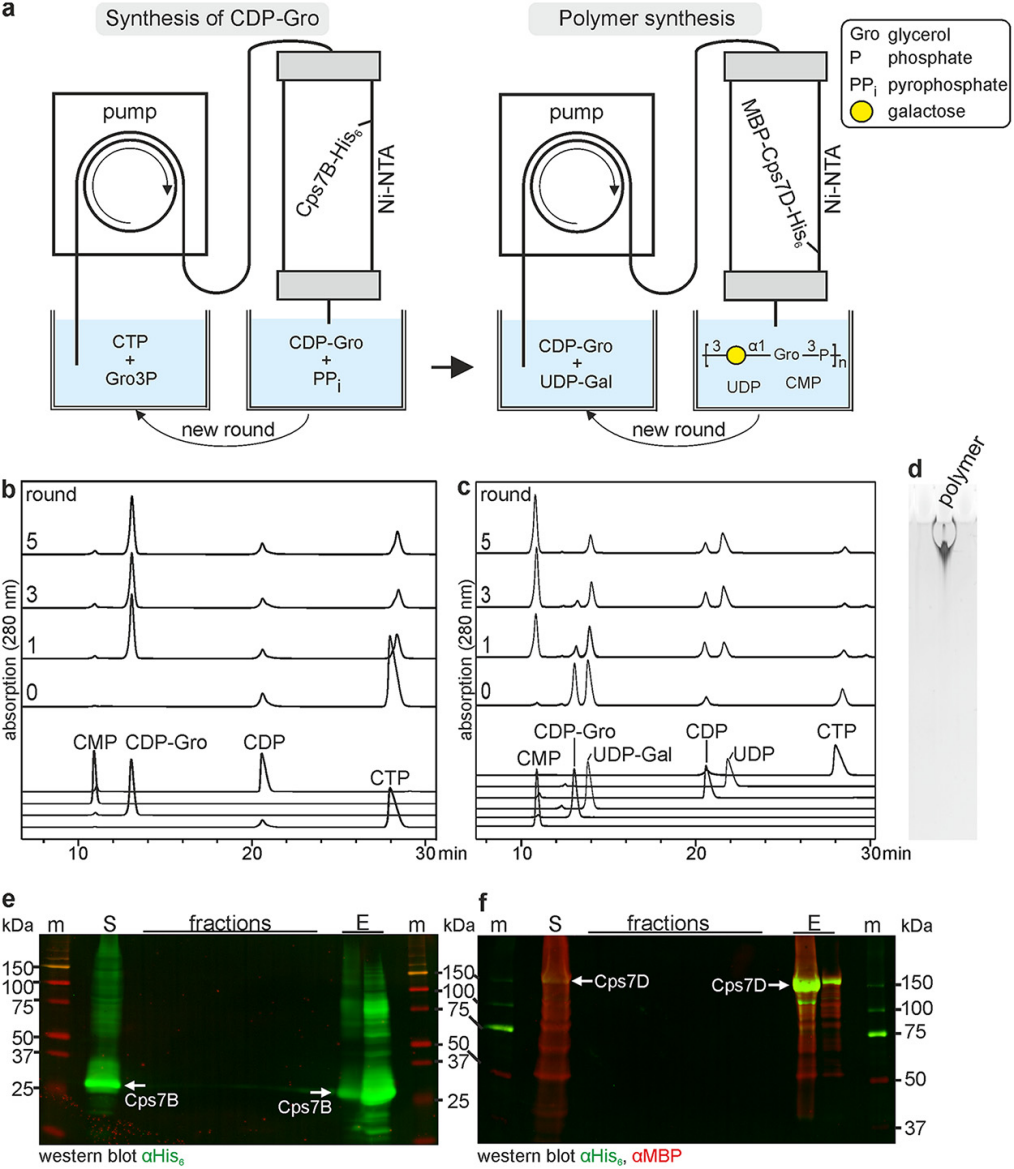
Solid-phase synthesis of CDP-Gro and enantiopure *App*7 polymer. (a) Schematic overview of the experimental setup. (b and c) HPLC-AEC analysis of the reaction mix before (0) and after the first (1), third (3) and fifth (5) round of circulation through Cps7B-loaded (b) and Cps7D-loaded (c) columns. (d) Alcian blue/silver-stained PA gel of polymer synthesized after round 5. (e and f) Western blot analysis of samples taken during CDP-Gro synthesis (e) and polymer synthesis (f). M, marker; S, supernatant; E, eluted protein; MBP, maltose-binding protein.

Recently, our group established protocols for the tailored, enzymatic solid-phase synthesis of capsule polymers from Neisseria meningitidis serogroups A and X (31) and A. pleuropneumoniae serotype 1 (32). Here, we apply this approach to Cps7B and Cps7D (Fig. 6a). The proteins Cps7B-His_6_ and MBP-Cps7D-His_6_ (MBP [maltose-binding protein]) were expressed in E. coli and separately immobilized on Ni-NTA columns via their C-terminal His_6_ tag. A reaction mixture containing CTP and Gro3P was circulated through the setup for several rounds (Fig. 6a, left), before the flowthrough was supplemented with UDP-Gal and transferred to the setup containing the Cps7D-loaded column (Fig. 6a, right). Samples were taken frequently to analyze reaction progress (Fig. 6b and c). As expected, immobilized Cps7B-His_6_ and MBP-Cps7D-His_6_ were active, as indicated by CDP-Gro (Fig. 6b), CMP/UDP (Fig. 6c), and polymer (Fig. 6d) production. Remaining UDP-Gal suggests a slight imbalance in the concentration ratio of both donor substrates (Fig. 6c). Small amounts of protein were detected in the collected fractions using Coomassie blue-stained SDS-PAGE (data not shown), and Western blot analyses demonstrate faint traces of free Cps7B-His_6_ during CDP-Gro synthesis (Fig. 6e). However, these traces seem to be retained by the Cps7D-loaded Ni-NTA column, since they disappear in the fractions collected during polymer synthesis (Fig. 6f). Both constructs could be eluted with imidazole from their respective columns at the end of the experiment (see fraction E in Fig. 6e and f), emphasizing stable binding during the course of the experiment and indicating that solid-phase synthesis protocols are generally achievable using both catalysts.

### *In situ* CDP-Gro synthesis starting from UTP and glycerol.

**FIG 7:**
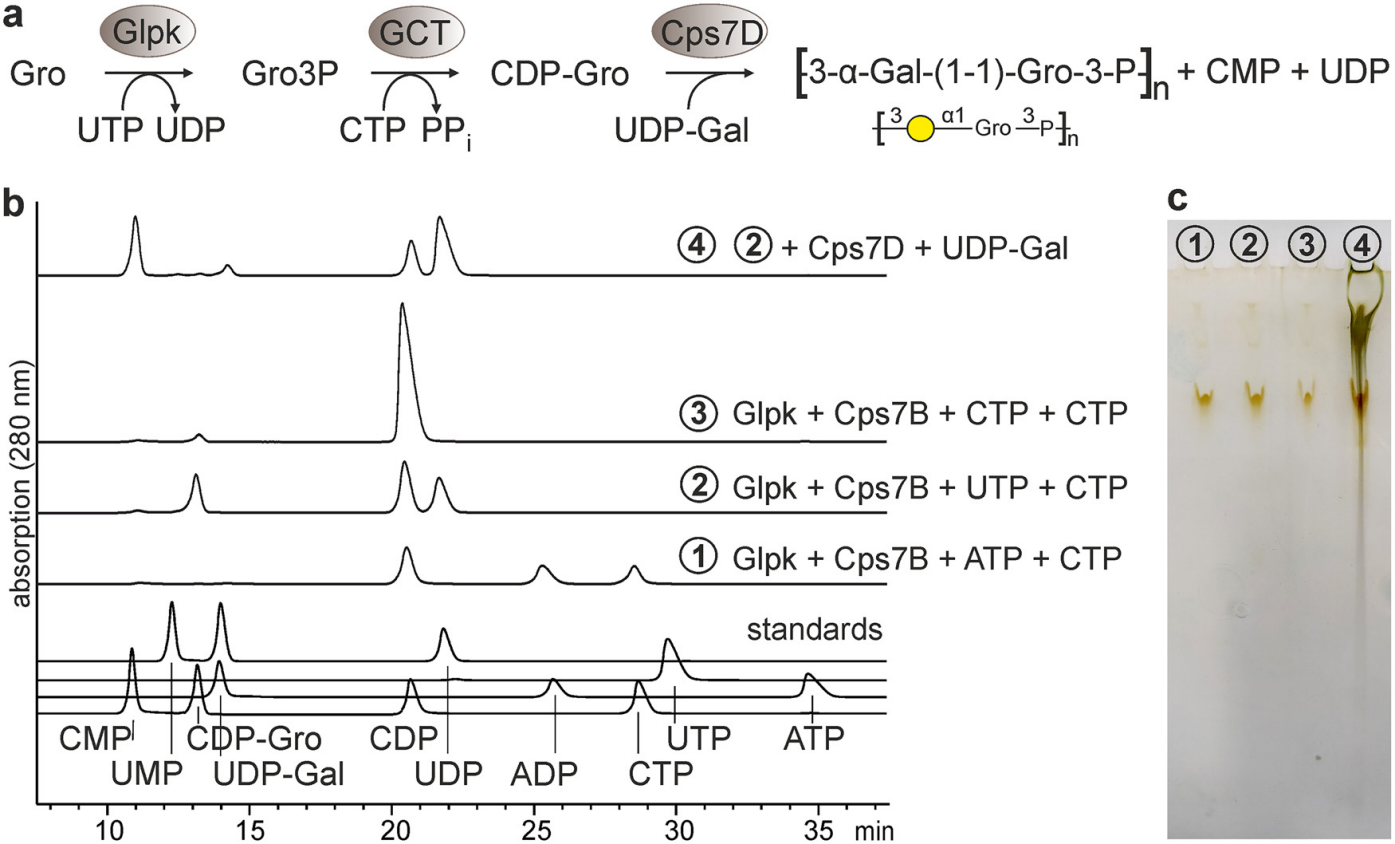
One-pot polymer synthesis with *in situ* provision of CDP-Gro starting from UTP and glycerol. (a) Scheme of the enzymatic cascade showing all enzymes and substrates. (b) HPLC-AEC analysis of one-pot reaction mixtures containing enzymes and substrates, as indicated. (c) Corresponding Alcian blue/silver-stained PA gel. Bands visible in all reactions result from silver-stained enzymes.

The broad availability of vaccines is highly dependent on production costs (10). To evaluate whether the synthesis cascade could be started from the inexpensive, widely available substrates glycerol and nucleoside triphosphates (Fig. 7a), the putative glycerol kinase (Glpk; putative ATP:glycerol-3-phosphotransferase) (33) from *App*7 was cloned, expressed, purified, and tested in combination with the GCT Cps7B. We considered ATP, UTP, and CTP as putative donor substrates for *App*7-Glpk. Substrate uptake and CDP-Gro formation were analyzed by HPLC-AEC. Interestingly, most CDP-Gro was produced in the presence of UTP (Fig. 7b, reaction 2), even though the majority of characterized Glpks exhibit a preference for ATP (34). ATP and CTP were converted to ADP and CDP, respectively, but CDP-Gro levels were not detectable or low (Fig. 7b, reactions 1 and 3). This observation indicates that these nucleoside triphosphates were subject to enzyme facilitated hydrolysis but are not suitable substrates for *in situ* CDP-Gro production in the enzyme cascade. In a next step, we performed a one-pot reaction mixture containing Glpk, the GCT Cps7B and the polymerase Cps7D, together with their substrates glycerol, UTP, CTP, and UDP-Gal, to enable the production of polymer (see reaction scheme in Fig. 7a). As expected, the polymerase substrates UDP-Gal and CDP-Gro were converted into the products CMP, UDP (Fig. 7b, reaction 4) and polymer (Fig. 7c, reaction 4), demonstrating that the *in situ* synthesis of CDP-Gro is possible starting from glycerol and UTP.

## DISCUSSION

This report presents the *in vitro* enzymatic production of complex glycerol-phosphate-containing group 2 capsule polymers from different Gram-negative bacteria. The initial focus was on the synthesis of nature-identical, enantiopure *App*3 and *App*7 capsule polymer backbones, which are assembled by the TagF-like polymerases Cps3D and Cps7D, respectively. To supply the commercially unavailable donor substrate CDP-Gro, the first aim was to establish an affordable and scalable CDP-Gro supply using the two putative GCTs Cps3B and Cps7B from *App*3 and *App*7, respectively. Although Cps3B and Cps7B utilized both commercially available GroP enantiomers, it is unlikely that both precursors are present in A. pleuropneumoniae. *In vivo*, GroP is produced by the glycerol phosphate dehydrogenase (GPDH) from achiral dihydroxyacetone phosphate (DHAP), a central metabolite of glycolysis (35). In *Bacteria* (and *Eukarya*), this enzyme synthesizes Gro3P, whereas in *Archaea*, Gro1P is produced (35). As an exception from this rule, a GPDH (AraM) specific for Gro1P was recently identified in B. subtilis and shown to be involved in the generation of an archaea-type ether lipid (36, 37). However, the fact that the lipid synthetic pathway is restricted to the *Bacillales* suggests a single horizontal gene transfer event from an archaeal species rather than an ubiquitous occurrence of AraM in bacteria (37). A BLAST search against the A. pleuropneumoniae proteome using the AraM amino acid sequence as the query yielded a single hit with 45% sequence identity. The identified enzyme, a putative 3-dehydroquinate synthase, is part of the shikimate pathway for the synthesis of aromatic amino acids, and is not known to harbor a GPDH activity (38).

Alternatively, GroP can be produced by glycerol kinase (Glpk), as presented here using Glpk from *App*7. Glpk is expressed in bacteria and heterotrophic archaea to allow the catabolic use of glycerol. To the best of our knowledge, it exclusively synthesizes Gro3P (39–41). Thus, it is reasonable to assume that free Gro1P is absent in the vast majority of bacteria. As a result, most studies assayed GCTs only in the presence of Gro3P. Only Rodrigues et al. (20) tested the GCTs AQ185 and AQ1368 from the Gram-negative bacterium Aquifex aeolicus, as well as one as-yet-unidentified GCT activity from lysates of the archaeon Archaeoglobus fulgidus, in the presence of both substrates. These researchers found AQ185 to be specific for Gro3P, whereas AQ1368 and the *A. fulgidus* enzymes utilized both substrates but showed a preference for Gro3P and Gro1P, respectively. Interestingly, the Gro3P-specific AQ185 belongs to the flavin adenine dinucleotide synthetase family. In contrast, the promiscuous AQ1368, like Cps3B and Cps7B, is part of to the cytidylyltransferase family. It is of note that the substrate ambiguity observed here makes Cps3B and Cps7B not only usable for the synthesis of Gro3P-containing bacterial structures but also for enzymatic reactions requiring CDP-1-Gro, e.g., during biosynthesis of archaean glycerophosphoinositol (20).

Despite the likely absence of CDP-1-Gro in bacteria, Gro1P has been found in surface structures such as lipoteichoic acid, phosphatidylglycerol, streptococcal rhamnose polysaccharides, and lipopolysaccharide (42–44). However, in all of these molecules the Gro1P moiety appears to originate from substrates other than CDP-1-Gro. For instance, during lipoteichoic acid synthesis, phosphatidylglycerol is formed after transfer of Gro3P and subsequent release of the sn-3-phosphoryl group from the resulting phosphatidylglycerol phosphate, retaining a Gro1P moiety that subsequently is transferred by the lipoteichoic acid synthase (45).

Similar to Cps3B and Cps7B, the polymerases Cps3D and Cps7D were able to utilize both GroP enantiomers from a racemic mixture of CDP-Gro, generating a mixed, artificial polymer (11). However, in the present study it became clear that only Cps7D can build a polymer from enantiopure CDP-1-Gro, although the natural polymer exclusively contains Gro3P (12). We hypothesize that this promiscuity is possible because in the *App*7 polymer the glycosidic linkage is formed via O1 of glycerol, which is two bonds away from the stereocenter at C2 (Fig. 1b). Interestingly, like Cps7D, the mammalian enzymes fukutin and fukutin-related protein that sequentially transfer two ribitol-phosphates during the synthesis of the laminin-binding epitope of *O-*mannosyl glycan, were also shown to accept CDP-1-Gro and CDP-3-Gro as the substrate (46). However, in this case, CDP-1-Gro was the better donor, presumably because the orientation of the hydroxyl group at the second carbon proximal to the phosphate group is the same in CDP-ribitol and CDP-1-Gro, but not in CDP-3-Gro (46). In contrast to Cps7D, Cps3D forms the glycosidic linkage directly with O2 at the stereocenter and was inactive in the presence of pure CDP-1-Gro. This finding clearly identifies CDP-3-Gro as the only possible substrate for Cps3D and additionally clarifies the stereochemistry of GroP in the *App*3 capsular polysaccharide, which had not been elucidated so far (13).

The efficacy of A. pleuropneumoniae glycoconjugate vaccines could be shown in initial studies (47–49). However, these vaccines never came to market, presumably due to the high production costs associated with obtaining the polymer from pathogen cultures. To reduce production costs and improve the lot consistency and homogeneity of glycoconjugate vaccines in general, considerable effort has been made to develop alternative means for antigen provision, e.g., by chemical synthesis (50), *in vitro* (chemo)enzymatic synthesis (51), and *in vivo* enzymatic synthesis in engineered E. coli safety strains (52, 53). The construction of enzymatic synthesis routes and the detailed understanding of the biocatalysts involved is crucial for the latter two strategies. An advantage of enzymatic synthesis is that polymer assembly starts from highly pure substrates, which simplifies downstream processing. Importantly, TagF-like polymerases only require nucleotide-activated donor substrates to initiate polymerization, omitting the need to provide acceptor saccharides (11). Here, we developed two separate enzymatic approaches for *in vitro* polymer production: one-pot and solid-phase synthesis. Although both methods are scalable and attractive for biotechnological use, only solid-phase synthesis can allow the reuse of enzyme-loaded matrices and does not require separation of the biocatalysts from the final products (54, 55). To further reduce production costs and increase scalability, the enzymatic cascade was expanded to include Glpk and GCT (Fig. 7), allowing the synthesis of enantiopure CDP-3-Gro from inexpensive starting materials that are widely available in large quantities.

**FIG 8:**
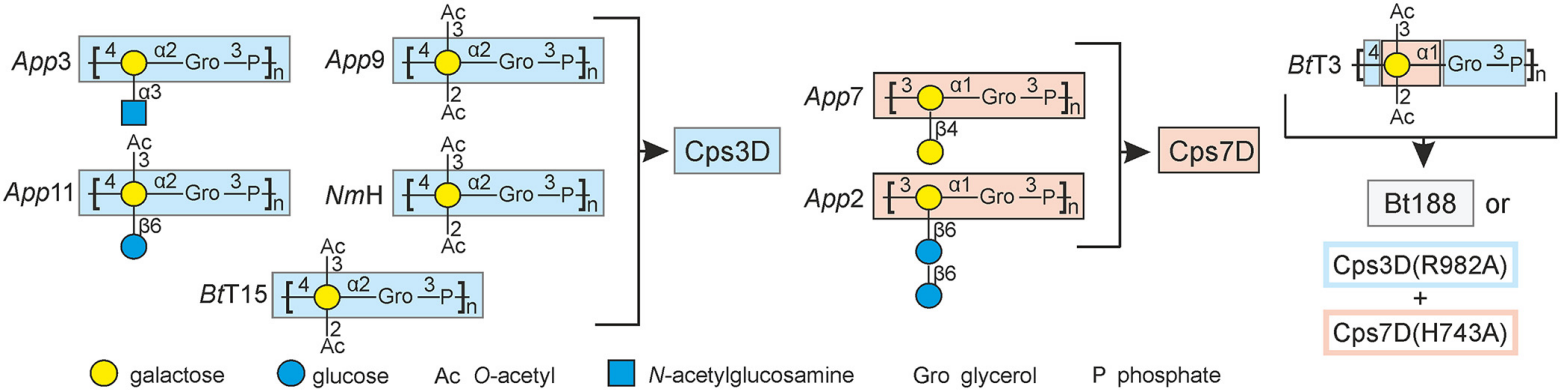
Overview of capsule polymer backbones producible with enzyme-based protocols described in this study. The native polymer structures are described in the following references: *App*3 (13), *App*9 (70), *App*11 (71), *Nm*H (72), *Bt*T15 (73), *App*7 (12), *App*2 (74), and *Bt*T3 (30).

Due to the fact that several pathogens express identical poly(glycosylglycerol phosphate) capsule polymer backbones, Cps3D and Cps7D can be used to supply polymer antigens for A. pleuropneumoniae serotypes 2, 3, 7, 9, and 11; B. trehalosi serotype T15; and Neisseria meningitidis serogroup H (*Nm*H) (Fig. 8). However, it is important to note that *in vivo*, these capsule polymer backbones are modified (12, 13). In group 2 capsule biosynthesis systems, such modifications are usually introduced by separate enzymes after assembly and, depending on the pathogen, can be beneficial or detrimental for the immunological response induced by a glycoconjugate vaccine (56, 57). Although a few group 2 capsule-modifying *O*-acetyltransferases have been identified and characterized (58, 59), group 2 capsule-modifying glycosyltransferases are still unknown. Future studies will show if these enzymes can be integrated in the synthesis cascade and if the modifications are necessary for a functional A. pleuropneumoniae vaccine.

A recent publication (60) authored by vaccine manufacturers, regulatory authorities, research institutions, and independent researchers in the field of vaccine development defined the challenges for future glycoconjugate vaccine development and identified the inadequate understanding of the mechanisms by which the immune system interacts with these complex antigens as a major drawback for vaccine improvement. The authors emphasized the importance of developing economic production platforms and encouraged studies that assess how factors like oligosaccharide size and structure impact the induced immune response. The technology presented here can contribute to both points, since the avoidance of pathogen culture and less laborious polymer purification can reduce costs. Moreover, previous studies have shown that the two-domain architecture of TagF-like polymerases can be exploited for the sequential buildup of tailored oligosaccharides, if single-action transferases are immobilized on separate solid phases (32). The authors further discussed that the knowledge gained from studying glycoconjugate vaccines in surrogate animal models is limited and often not transferable to the immune response in humans, making it hard to deduce a mechanism of action for these vaccines in general (60). Glycoconjugate vaccines against, e.g., A. pleuropneumoniae, can be studied in pigs as their natural host organism, omitting the use of a surrogate animal model and allowing us to draw more accurate conclusions from studies analyzing how certain vaccine characteristics (e.g., carrier protein, chain length, and coupling chemistry) influence the induced immune response and how protection against the pathogen is achieved. Thus, the study of anti-A. pleuropneumoniae glycoconjugate vaccines made with the technology presented here might not only improve our knowledge of the still poorly understood cell-mediated immune responses against an A. pleuropneumoniae infection (4, 61) but can also contribute to a better understanding of the mechanism of action of glycoconjugate vaccines in general.

The value of easily adaptable vaccine production platforms that are pathogen-free and allow quick responses to novel diseases is impressively demonstrated by the current COVID-19 pandemic (62). We present here a highly versatile toolbox for antigen synthesis. This is ideal for vaccines against A. pleuropneumoniae, since the predominance of A. pleuropneumoniae serotypes can vary geographically and temporally (4), increasing the necessity for frequent adaptation of the vaccine antigen. The versatility of the presented platform can be even further increased by exploiting the modular architecture of TagF-like polymerases, which allows the creation of single-action transferases that in turn can be combined in *trans* with other single-action transferases to generate entirely new polymer structures. This tempts us to speculate that TagF-like polymerases might be exploitable for the (chemo)enzymatic synthesis of other phosphate-containing structures like Gram-positive wall teichoic acids or, due to the substrate promiscuity of Cps3B/7B and Cps7D, even for Gro1P-containing lipoteichoic acids and O antigens.

## MATERIALS AND METHODS

### Bioinformatics.

BLASTP (63) (protein-protein BLAST) searches were performed against the nonredundant protein sequence database. The sequence alignment was performed with Clustal Omega (64) using the UniProt website (http://www.uniprot.org/align/) (65) and annotated with Jalview (66). Bruker TopSpin version 4.0.7 was used to process NMR data. Sparky (T. D. Goddard and D. G. Kneller, SPARKY 3; University of California, San Francisco) was used to analyze and assign NMR data.

### General cloning.

All primers and restriction enzymes used in this study are listed in Tables S1 and S2 in the supplemental material. *cps3B*/*cps7B* and *cps11D* were amplified by PCR from heat-inactivated bacterial lysates (Table 2) and cloned into plasmids *p*ΔN37-cslB-His_6_ (25) and *p*MBP-csxA-His_6_ (67), respectively, replacing *cslB* and *csxA*, resulting in the constructs *p*cps3B-His_6_/*p*cps7B-His_6_ and *p*MBP-cps11D-His_6_. The construction of Cps3D, Cps7D, and Cps7D (H743A) was described previously (11). The Bt188 amino acid sequence (GenBank AHG82487.1) was reverse translated into DNA (see Text S1 in the supplemental material), codon optimized for expression in E. coli, synthesized by General Biosystems, and cloned via NdeI/XhoI into expression vector pET-22b(+), resulting in the plasmid *p*Bt188-His_6_. Single-amino-acid mutations in Cps3B and Cps3D were introduced using the Q5 site-directed mutagenesis kit (New England Biolabs) according to the manufacturer’s guideline, and plasmids *p*cps3B-His_6_ and *p*MBP-cps3D-His_6_ (11) were used as the template, respectively. *Glpk* was amplified via PCR from heat-inactivated bacterial lysate (Table 2) and cloned via restriction-free cloning (68) into plasmid *p*cps7B-His_6_, replacing *cps7B*.

**TABLE 2:** Strains and enzymes used in this study

Strain	Serotype	Protein	Accession no.
Actinobacillus pleuropneumoniae S1421	*App*3	Cps3B	GenBank, ABY70165.1
Actinobacillus pleuropneumoniae AP76	*App*7	Cps7B	GenBank, ACE62293.1
Actinobacillus pleuropneumoniae S1421	*App*3	Cps3D	GenBank, KY807157
Actinobacillus pleuropneumoniae AP76	*App*7	Cps7D	GenBank, ACE62291.1
Actinobacillus pleuropneumoniae 56153^*a*^	*App*11	Cps11D	UniParc, UPI0001E49633
Nonserotyped Bibersteinia trehalosi strain USDA-ARS-USMARC-188^*b*^		Bt188	GenBank, AHG82487.1
Actinobacillus pleuropneumoniae AP76	*App*7	Glpk	GenBank, ACE61051

a Frey and Nicolet (75), Xu et al. (15).

b Harhay et al. (26).

10.1128/mBio.00897-21.1 TEXT S1 Codon-optimized Bt188 DNA sequence. Restriction sites are underlined. Download Text S1, PDF file, 0.2 MB. Copyright © 2021 Litschko et al. 2021 Litschko et al. https://creativecommons.org/licenses/by/4.0/ This content is distributed under the terms of the Creative Commons Attribution 4.0 International license.

10.1128/mBio.00897-21.3 TABLE S1 Strains, enzymes, and recombinant constructs generated and used in this study. Download Table S1, PDF file, 0.3 MB. Copyright © 2021 Litschko et al. 2021 Litschko et al. https://creativecommons.org/licenses/by/4.0/ This content is distributed under the terms of the Creative Commons Attribution 4.0 International license.

10.1128/mBio.00897-21.4 TABLE S2 Primers used in this study. Restriction sites are underlined. Download Table S2, PDF file, 0.2 MB. Copyright © 2021 Litschko et al. 2021 Litschko et al. https://creativecommons.org/licenses/by/4.0/ This content is distributed under the terms of the Creative Commons Attribution 4.0 International license.

### Expression and purification of recombinant proteins.

Expression and purification of Cps3D, Cps7D, Cps11D, Bt188, Cps3D(R982A), and Cps7D(H743A) were performed as described previously (11, 25). Cps3B, Cps7B, all Cps3B mutants, and Glpk were purified accordingly, with the adjustment that the buffer contained 10 mM Tris-HCl (pH 8.0), 300 mM NaCl, and 10% glycerol (+ 500 mM imidazole for elution). The protein yield was determined spectrophotometrically using the theoretical extinction coefficients predicted by ProtParam (69).

### Enzymatic reactions, HPLC-AEC analysis, and high-percentage polyacrylamide gel electrophoresis.

All enzymatic reactions were carried out with 1 to 2 μM enzyme at 37°C in a total volume of 25 to 50 μl of assay buffer (20 mM Tris-HCl [pH 8.0], 1 mM DTT [dithiothreitol], 10 mM MgCl_2_). The substrate concentration of CTP (Sigma), Gro1P and Gro3P (both Sigma), and, if required, UDP-Gal (Carbosynth) was 5 mM. In reaction mixtures containing Glpk, 5 mM substrates (CTP and ATP [Roche] and UTP [Sigma]) were used, and pyrophosphatase (0.003 U/μl; Invitrogen) was added. Glycerol was supplied from the enzymes’ storage buffer, yielding 0.7% glycerol in the final reaction mixture. Samples for the high-performance liquid chromatography (HPLC) and PAGE analyses were taken prior to the addition of enzymes and after 2 h of incubation or after an overnight reaction. High-performance liquid chromatography–anion-exchange chromatography (HPLC-AEC) and PAGE analyses were performed as described previously (11, 29) with minor adjustments: The chromatograms shown in Fig. 2 and 3 were recorded using a linear elution gradient from 0 to 25% of mobile phase 2 over 14 min. The chromatograms shown in Fig. 2d and Fig. 4 to 7 were recorded with a linear elution gradient from 0 to 25% of mobile phase 2 over 42 min and 5 μl of sample.

### Upscaling of polymer synthesis and purification.

Polymer (5 mg) was synthesized in one-pot reactions by combining GCTs and polymerases in reaction buffer (20 mM Tris-HCl [pH 8.0], 10 mM MgCl_2_, 1 mM DTT) supplemented with 5 mM concentrations of each donor substrate in a total volume of 3.2 ml at 37°C overnight. The amount of GCT ranged from 7.5 to 28 nmol, and 7.5 to 9 nmol of polymerase was used. Polymer was purified by AEC as described previously (11).

### NMR analyses.

NMR data were recorded on a Bruker Avance III HD 600-MHz spectrometer with a QXI room-temperature probe for ^1^H/^13^C/^15^N/^31^P (Bruker Biospin, Germany) at 298 K in D_2_O as described previously (11).

### Solid-phase synthesis.

Expression of Cps7B and Cps7D was performed as described previously (11), solid-phase synthesis was performed according to published protocols (31, 32) with minor adjustments: 250 ml of E. coli M15 (pRep4) expression culture was used for coupling of enzymes to 1 ml Ni-NTA columns (Cytiva). The Cps7B-loaded column was thoroughly washed with binding buffer A (10 mM Tris-HCl [pH 8.0], 300 mM NaCl, 10% glycerol, 1 mM DTT). The Cps7D-loaded column was thoroughly washed with binding buffer B (50 mM Tris-HCl [pH 8.0], 500 mM NaCl, 1 mM DTT). Both columns were equilibrated in 20 mM Tris-HCl (pH 8.0), 10 mM MgCl_2_, and 1 mM DTT. Portions (5 ml) of the reaction mixture (equilibration buffer containing 6 mM CTP and 6 mM Gro3P) were circulated at a flow rate of 0.4 ml/min through a Cps7B-loaded column. After several rounds of circulation, the flowthrough was supplemented with 5 mM UDP-Gal and circulated through the Cps7D-loaded column (0.4 ml/min). Fractions were collected as indicated (Fig. 6). Recovery of enzymes was achieved using elution buffer (binding buffer A supplemented with 500 mM imidazole for Cps7B and binding buffer B supplemented with 500 mM imidazole for Cps7D). Samples were analyzed by using HPLC-AEC and Alcian blue/silver-stained PAGE as described above. Samples from Cps7B- and Cps7D-loaded columns were separated by SDS-PAGE (16 and 10%, respectively) and analyzed by Western immunoblotting as described previously (67) using anti-penta-His (Qiagen) and anti-MBP (antibodies-online) as primary antibodies and goat anti-mouse IR800 (LI-COR) and goat anti-rabbit IR680 (LI-COR) as secondary antibodies. Western blot bands were detected with the Odyssey infrared imaging system (LI-COR).

### Data availability.

All data are presented in the manuscript or supplemental material, including NMR chemical shifts (Table 1). Raw NMR data are available from the corresponding author upon request.
