# Supplementary material for: Mix-and-Match System for the Enzymatic Synthesis of Enantiopure Glycerol-3-Phosphate-Containing Capsule Polymer Backbones from Actinobacillus pleuropneumoniae, Neisseria meningitidis, and Bibersteinia trehalosi
Source: mBio. 2021 May 26;12(3):e00897-21. doi: 10.1128/mBio.00897-21 (PMC8262930; doi:10.1128/mBio.00897-21)
Supplement: FIG S1 [file mbio.00897-21-sf001.pdf]

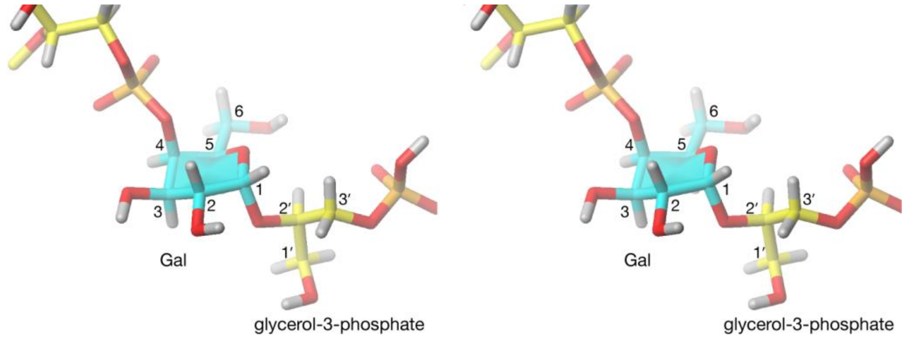

Fig. S1. Stereoview of a repeating unit of a [→4)-α-Gal-(1→2)-Gro-(3-PO_4^-] (*App*3) hexamer. The distances between Gal H1 and the surrounding protons in the model are in good agreement to the distances derived from NOE cross-peak intensities from 2D NOESY spectra shown in Fig. 3g (H1-H2’, H1-H3a’, H1-H3b’ all < 3.0 Å) according to (Aeschbacher T, Zierke M, Smieško M, Collot M, Mallet J-M, Ernst B, Allain FHT, Schubert M. 2017, Chemistry, 23:11598–11610, doi: 10.1002/chem.201701866). The model was generated using CarbBuilder (Kuttel MM, Ståhle J, Widmalm G. 2016, J Comput Chem, 37:2098–105, doi: 10.1002/jcc.24428), was further refined by a molecular dynamics calculation using Yasara (Krieger E, Vriend G. 2015, J Comput Chem, 36:996–1007, doi: 10.1002/jcc.23899) and the image was generated using Molmol (Koradi R, Billeter M, Wüthrich K. 1996, J Mol Graph, 14:51–5, 29–32, doi: 10.1016/0263-7855(96)00009-4).
