# Supplementary material for: Mix-and-Match System for the Enzymatic Synthesis of Enantiopure Glycerol-3-Phosphate-Containing Capsule Polymer Backbones from Actinobacillus pleuropneumoniae, Neisseria meningitidis, and Bibersteinia trehalosi
Source: mBio. 2021 May 26;12(3):e00897-21. doi: 10.1128/mBio.00897-21 (PMC8262930; doi:10.1128/mBio.00897-21)
Supplement: TEXT S1 [file mbio.00897-21-s0001.pdf]

Supplemental Text S1. Codon-optimized Bt188 DNA sequence. Restriction sites are underlined.

CATATGAAGTACAAGGTGAAACTGAGCCTGATTCCGATGCTGCTGAAAGGCCTGTATGCCTATAAACATAAAAATTATAACGAGGCCGA
GCGTATTTTTGCACTGCTGCTGGATAAACAGGGCGATAATGCCTATCTGAATTTTCGCTATGGCATGGCCCTGTATAAAAGTAAAAAATG
GGATGAAGCCAACTATTATATCCAGAAAGCAATTAATATCGACCCGAGTAAAAAAAGTTGGCAGGTGCAGCTGGCAACCAGCGAACGTT
ATAAACAGAATAGTGAAAAAGTGAAGGTGGCAGAAGCAAAAAAAGCAGCAATTAAAGATCCGAATAGTCCGGAAGTTATTTGGGAATA
TGCAATTAGTCTGCTGGAAAATAAACAGTATTGGATGGCCAAACTGCAGTTTGAAAAATATCTGATTCTGAAACCGAACAGCGAAAAAG
CATTTGATAATCTGGGTAAAGTTAGTGAAAAGCTGAGTGATTATCCGGAAGCCATTAAATATTTTGAAAAGGCAGCAAGTCTGAGCCCGT
TTAATAGCAATTATAAATATCGCATCGGCTATAGTTACGAATGTCTGGGCGATCTGGAAAATGCCAATACCTATTATGAACTGGTTAGCA
GCTTTGCCAAAAGCAATGATGATGTGGCACTGTTTGGCATTGGCGCACTGCATGCAAAACGTGGCCTGTGGGATAAAGCACTGAATGCA
TATAAACAGTTTCTGGCAAAAACCCAGAGTAATAATCCGGAACTGTATTATCGTATTGGTGTTGCAAATGAACGCCTGTATCTGTGGGAA
GATGCCGGCAAAGCATTTGAACAGGCCGTTAAACTGAGTGAAATTATTACCGCACCGTGGTGTTTTAAATGTGGCCAGGCATACGAACG
CGCCAAAAATTATGCCAAAGCCGTTGAATTTTACCAGAATGCAGTGGCACGCAGTAATAATTATAATGATTATTGGCTGTACCGCCTGGC
CTATAGTATGGAAATGATGGGTAATTTTGAGCAGGCCGCAAAATATTATCAGCAGAGTCGCCGTCGCAAAATTGCCCATGCAGTTGCACC
GAAAGATGTGATTAAAAATAAAGAAGAGGAGTACCTGACCTATTATACCGAATATTATGAAACCCTGGCCGTGAATGAAAAACAGGTTC
TGATTGAAAGTTTCTTTGGTGGTAATATTAGCTGTAATCCGTATGCAATTCTGCTGTATATGCTGGATCATAATTATGATTTCACCTATATC
GTGGTTGTTAAACCGGAAACCGTTATTCCGGATAGCCTGAAATTTAAACAGAATATTATCTTCATCAACCGCGGCAGTGATGCCTATCTGC
GCTATCTGTGTACCGCAAAATATCTGATCAATAATGTTAGTTTCCCGTATTATTTCATCCGCAAAGCCGAACAGATTTATCTGAATACCTGG
CATGGTACCCCGATGAAAACCCTGGGTAAAGATATTAAAAGTCCGTTTCAGGATCATAGTAATGTTAGTCGTAATTTTCTGCAGGCAACC
CATCTGATTAGCCCGAATCGCCATACCACCGATATTATGCTGGAAAAATATGATATCAAGGACCTGTTTAGTGGTGAAATTGCAGAAACC
GGTTATCCGCGTATTGATCTGAGCTTTCTGAGTGAAGAACGCCGCAATGAAATTCGTAAAAAACTGGGTTTTAAGAACAACAAACCGGTG
GTTTTTTATGCCCCGACCTGGCGTGGCACCAGCCAGAGTAAAGATTTTGATACCCAGAAACTGCAGAATGATCTGAAACGCCTGAAAAGC
GATAAATATAATCTGGTGTTTCGCGGCCATCATCTGGTGGAAAGCCTGCTGAGCGAAATTAAACTGGATGTTGTTGTTGCACCGAAGGA
AATTGATAGCAATGAACTGCTGGGTTATTGCGATCTGCTGATTACCGATTATAGTAGCATTATTTACGACTTTCTGGCACTGAATAAACCG
GTTATTAGCTATGTTTATGACTTTGATGAGTACAAAGAGGAACGTGGTCTGTATTTTGAAAAAGATGAAATGGTTGGCGCCGTTTGTAGC
ACCATTAGTGAAGTGCGTCAGGCCATTCTGGAAAATCTGAATAAAAATAAGAGCAACGTGCTGGAACGTGATATTGAAAAATATAGTTA
CCTGGATGACGGCCGTGCAACCCAGCGCACCGTTGATTTTATTTTTAAAAATGACAACCGTTACGTGTACGATTATATTCGTAAAGATACC
GATGTTTTCTTCGTGGGTCCGTTTCTGCAGAATGGTATTACCCGTAGCTTTCTGAATCTGATGAGCACCATTGGTCGCGAAAAAAATATTC
TGGTGCTGATTAATGGCAATGATCTGCAGAGTGATAATAAACGTCTGGAAGAATTTTACCGTCTGCCGAAAGATATTAGTGTTTTTAGCC
GCAGTGGCCGCATGCTGATGACCCTGGAAGAACTGTGGGTGCGCAATAAATTTGATGAAAATTTTAAGTTCTACAGCGAGGAATTTAAG
CGTGTGATTGAAAAAATTTACAAGCGCGAAGCACGCCGTCTGTTTGGTGATAGTAAAATTCGTAATATCATCAACTTCGAGGGCTATGCA
CTGTTTTGGGTTCTGCTGATTAGTCAGGTGAATGCCAAACAGCATATTATTTATCAGCATAATGACAAGTACAAGGAATGGAAAAGTAAA
TTTCCGTATCTGGAAGGTGTTTTTCGCACCTATCGTTATTATGATAAAATCGTTAGCGTGAGTGAAAAAACCATGGAAAATAATCGCAATA

ACATCAGTTACGAATTCGGTATTGCAGAAAAACGCTTTGTTTTTTGCAATAATCCGATTAATATCGATCAGATTATCAGCAATGCCAAAGA
TGATATTGAAATTGAAGACGAATTCGATAACTTCGCAGGTACCAAATTTATTAATATCGGCCGTATGAGTCATGAAAAAGATCAGCTGAA
ACTGATTGAAGCATTTGCAGAAGTTAATAAAAAGCATAAGGACACCCGCCTGTTTATTCTGGGCGATGGTCCGCTGAAACAGGAACTGA
TTACCCGTATTAAAAAACTGAGCTTAGAAAAAGACGTGTTTCTGCTGGGCCAGAAAACCAATCCGTTTGCATATCTGAAACAGGCAGATA
TTTTTGTGCTGAGTAGTAATCATGAAGGCCAGCCGATGGTTCTGCTGGAAAGCCTGACCCTGGGTACCCCGATTATTGCAACCGATATTG
TGGGCAATCGCAGCATTCTGGGCGACAAATATGGCCTGCTGGTTGAAAATAGCAAACAGGGTCTGATTAATGGTATGAATGAATATCTG
GAAAACGGCAGTAAACAGGATAATTTTGATCCGATTGCATATCAGAAAGATGCAATGGATAAATTTTACGCCCTGCTGAATGAACTCGA
G
