# Supplementary material for: Mix-and-Match System for the Enzymatic Synthesis of Enantiopure Glycerol-3-Phosphate-Containing Capsule Polymer Backbones from Actinobacillus pleuropneumoniae, Neisseria meningitidis, and Bibersteinia trehalosi
Source: mBio. 2021 May 26;12(3):e00897-21. doi: 10.1128/mBio.00897-21 (PMC8262930; doi:10.1128/mBio.00897-21)
Supplement: TABLE S1 [file mbio.00897-21-st001.pdf]

Supplemental Table S1

Strains, enzymes and recombinant constructs generated and used in this study.

Strain (Ref.)	Serotype	Protein	Accession number	Plasmid	Identifier	Recombinant construct	MW	Primer	Restriction site
Actinobacillus pleuropneumoniae S1421	*App*3	Cps3B	Genbank: ABY70165.1	*p*cps3B-His$_6$	5113	Cps3B-His$_6$	17.5 kDa	TF117 / TF116	NdeI/XhoI
				*p*cps3B-H14A-His$_6$	5715	Cps3B-H14A-His$_6$	17.5 kDa	CL243 / CL244	
				*p*cps3B-H17A-His$_6$	5716	Cps3B-H17A-His$_6$	17.5 kDa	CL245 / CL246	
				*p*cps3B-K44A-His$_6$	5717	Cps3B-K44A-His$_6$	17.5 kDa	CL247 / CL248	
				*p*cps3B-K46A-His$_6$	5718	Cps3B-K46A-His$_6$	17.5 kDa	CL249 / CL250	
				*p*cps3B-R113A-His$_6$	5719	Cps3B-R113A-His$_6$	17.5 kDa	CL251 / CL252	
		Cps3D	Genbank: KY807157	*p*MBP-cps3D- His$_6$	5360	(1)			
				*p*MBP-cps3D-R982A-His$_6$	5393	MBP-Cps3D-R982A-His$_6$	176 kDa	CL219 / CL220	
Actinobacillus pleuropneumoniae AP76	*App*7	Cps7B	Genbank: ACE62293.1	*p*cps7B-His$_6$	5152	Cps7B-His$_6$	17.5 kDa	TF117 / TF116	NdeI/XhoI
		Cps7D	Genbank: ACE62291.1	*p*MBP-cps7D-His$_6$	4887	(1)			
				*p*MBP-cps7D-H743A-His$_6$	5055	(1)			
		Glpk	Genbank: ACE61051	*p*glpk-His$_6$	5797	Glpk-His$_6$	57 kDa	TF180 / TF181	
Actinobacillus pleuropneumoniae 56153 (2, 3)	*App*11	Cps11D	UniParc: UPI0001E49633	*p*MBP-cps11D-His$_6$	5402	MBP-Cps11D-His$_6$	176 kDa	TF156 / TF157	BamHI / XhoI
non-serotyped *Bibersteinia trehalosi* strain USDA-ARS-USMARC-188 (4)		Bt188	GenBank: AHG82487.1	*p*Bt188-His$_6$	5419	Bt188-His$_6$	134 kDa	Synthesized (*General Biosystems*)	NdeI/XhoI

References

1. Litschko C, Oldrini D, Budde I, Berger M, Meens J, Gerardy-Schahn R, Berti F, Schubert M, Fiebig T. 2018. A New Family of Capsule Polymerases Generates Teichoic Acid-Like Capsule Polymers in Gram-Negative Pathogens. MBio 9:16017.
2. Xu Z, Chen X, Li L, Li T, Wang S, Chen H, Zhou R. 2010. Comparative genomic characterization of Actinobacillus pleuropneumoniae. J Bacteriol 192:5625–36.
3. Frey J, Nicolet J. 1990. Hemolysin patterns of Actinobacillus pleuropneumoniae. J Clin Microbiol 28:232–6.
4. Harhay GP, McVey DS, Koren S, Phillippy AM, Bono J, Harhay DM, Clawson ML, Heaton MP, Chitko-McKown CG, Korlach J, Smith TPL. 2014. Complete Closed Genome Sequences of Three Bibersteinia trehalosi Nasopharyngeal Isolates from Cattle with Shipping Fever. Genome Announc 2.
