# Supplementary material for: Mix-and-Match System for the Enzymatic Synthesis of Enantiopure Glycerol-3-Phosphate-Containing Capsule Polymer Backbones from Actinobacillus pleuropneumoniae, Neisseria meningitidis, and Bibersteinia trehalosi
Source: mBio. 2021 May 26;12(3):e00897-21. doi: 10.1128/mBio.00897-21 (PMC8262930; doi:10.1128/mBio.00897-21)
Supplement: TABLE S2 [file mbio.00897-21-st002.pdf]

Supplemental Table S2

Primers used in this study. Restriction sites are underlined.

Primer	Sequence
TF117	5’-GCATCTCATATGAAAAAAGTATTAACCTATGGAACC-3’
TF116	5’-GCATCTCTCGAGCTCTTTTTCGTGAATTTGTTTTTGTC-3’
TF156	5’-GCATCTGGATCCTTATTAAAAAGCGAGAACTTTAAAATGAAACATAATG-3’
TF157	5’-GCATCTCTCGAGATTTGTTAATAATGAATAAAACTTCGCCATAGC-3’
TF180	5’-ACGAGCACTTCACCAACAAGGACCATAGCATATGACTAAAGAATACATTATTGCGTTAGACCAAGG-3’
TF181	5’-TGGTGGTGGTGGTGGTGCTCGAGTTCCTCTGCGTCTTCTTTTGCCCATTC-3’
CL243	5’-TGATTTATTAGCACATGGGCATATTCGTTTATTAGAAAGAGCAAG-3’
CL244	5’-ATGCCCATGTGCTAATAAATCAAAGGTTCCATAGGTTAATAC-3’
CL245	5’-ACACCATGGGGCTATTCGTTTATTAGAAAGAGCAAGATCATTAG-3’
CL246	5’-TCTAATAAACGAATAGCCCCATGGTGTAATAAATCAAAGGTTCC-3’
CL247	5’-AACTTAGGAGCAGGCAAAGTATGCGCTTATACTTACGAAGAGAG-3’
CL248	5’-ACTTTGCCTGCTCCTAAGTTAAATTGATCGGTAGAAATAGCAACAG-3’
CL249	5’-AGGAAAAGGCGCAGTATGCGCTTATACTTACGAAGAGAGAGCG-3’
CL250	5’-AAGCGCATACTGCGCCTTTTCCTAAGTTAAATTGATCGGTAGAAATAGC-3’
CL251	5’-AGTTTATTTACCTGCAACTCCTGATATTTCAACCACTCAAG-3’
CL252	5’-ATCAGGAGTTGCAGGTAAATAAACTACTTCGCAATAATCTGCTAAG-3’
CL219	5’-AATATTGGCGCGATGTCGCAC-3’
CL220	5’-AATAAACTTCTGGCCATTGAATAAAGTAAACTC-3’
